# Pentacycloundecane lactam vs lactone norstatine type protease HIV inhibitors: binding energy calculations and DFT study

**DOI:** 10.1186/s12929-015-0115-5

**Published:** 2015-02-18

**Authors:** Bahareh Honarparvar, Sachin A Pawar, Cláudio Nahum Alves, Jerônimo Lameira, Glenn EM Maguire, José Rogério A Silva, Thavendran Govender, Hendrik G Kruger

**Affiliations:** Catalysis and Peptide Research Unit, School of Health Sciences, University of KwaZulu-Natal, Durban, 4041 South Africa; Laboratório de Planejamento e Desenvolvimento de Fármacos, Instituto de Ciências Exatas e Naturais, Universidade Federal do Pará, CP 11101, 66075-110 Belém, PA Brazil

**Keywords:** Pentacyclo-undecane (PCU), Lactone, Lactam and lactim peptides, Human immunodeficiency virus protease (HIV PR), Inhibitory concentration (IC_50_), Molecular dynamics (MD), MM-PB (GB) SA method

## Abstract

**Background:**

Novel pentacycloundecane (PCU)-lactone-CO-EAIS peptide inhibitors were designed, synthesized, and evaluated against wild-type C-South African (C-SA) HIV-1 protease. Three compounds are reported herein, two of which displayed IC_50_ values of less than 1.00 μM. A comparative MM-PB(GB)SA binding free energy of solvation values of PCU-lactam and lactone models and their enantiomers as well as the PCU-lactam-NH-EAIS and lactone-CO-EAIS peptide inhibitors and their corresponding diastereomers complexed with South African HIV protease (C-SA) was performed. This will enable us to rationalize the considerable difference between inhibitory concentration (IC_50_) of PCU-lactam-NH-EAIS and PCU-lactone-CO-EAIS peptides.

**Results:**

The PCU-lactam model exhibited more negative calculated binding free energies of solvation than the PCU-lactone model. The same trend was observed for the PCU-peptide inhibitors, which correspond to the experimental activities for the PCU-lactam-NH-EAIS peptide (IC_50_ = 0.076 μM) and the PCU-lactone-CO-EAIS peptide inhibitors (IC_50_ = 0.850 μM). Furthermore, a density functional theory (DFT) study on the natural atomic charges of the nitrogen and oxygen atoms of the three PCU-lactam, PCU-lactim and PCU-lactone models were performed using natural bond orbital (NBO) analysis. Electrostatic potential maps were also used to visualize the electron density around electron-rich regions. The asymmetry parameter (η) and quadrupole coupling constant (χ) values of the nitrogen and oxygen nuclei of the model compounds were calculated at the same level of theory. Electronic molecular properties including polarizability and electric dipole moments were also calculated and compared. The Gibbs theoretical free solvation energies of solvation (∆G_solv_) were also considered.

**Conclusions:**

A general trend is observed that the lactam species appears to have a larger negative charge distribution around the heteroatoms, larger quadrupole constant, dipole moment and better solvation energy, in comparison to the PCU-lactone model. It can be argued that these characteristics will ensure better eletronic interaction between the lactam and the receptor, corresponding to the observed HIV protease activities in terms of experimental IC_50_ data.

**Electronic supplementary material:**

The online version of this article (doi:10.1186/s12929-015-0115-5) contains supplementary material, which is available to authorized users.

## Background

In spite of extensive investigations and clinical efforts made over more than two decades on the extinction of HIV, AIDS is still a substantial threat to global health [1-7]. HIV treatment comprises an amalgam of a wide arsenal of various drugs that target different stages in the viral replication cycle. The majority of synthesized drugs for blocking viral enzymes, act as transcriptase (RT) and protease (PR) inhibitors [8].

**Figure 1:**
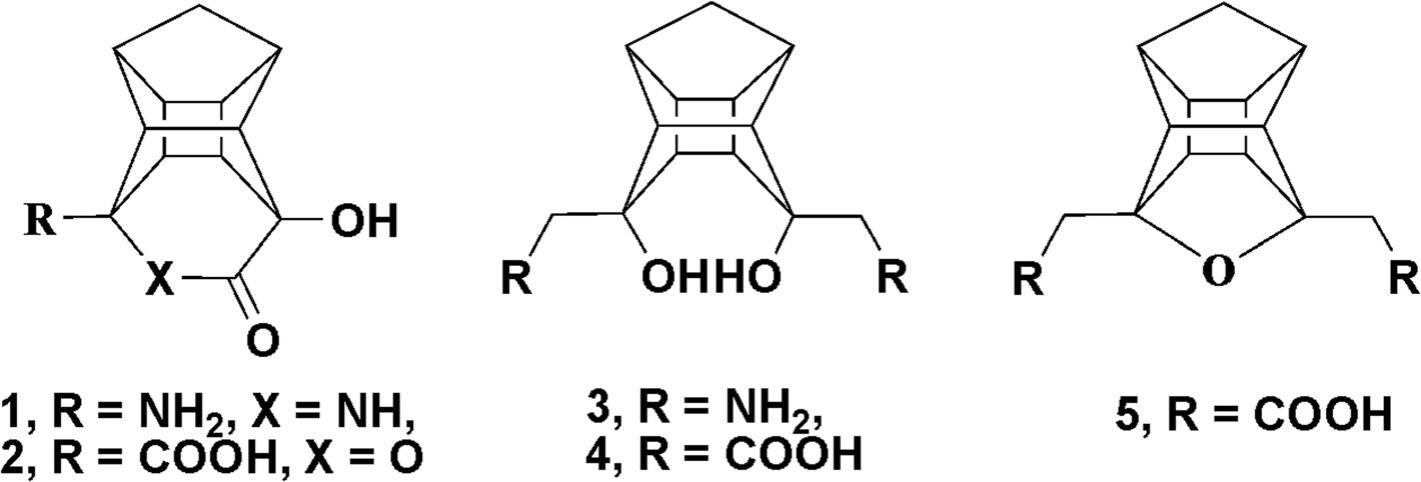
**Structures of different types of cages** [12-14,16]**.**

Lactams [9,10] and lactones [11] are well known anti-bacterial agents. Lactones exhibit high potency for the treatment of skin and soft tissue infections [11]. We have recently reported pentacycloundecane (PCU) derived cage peptides, functionalised as lactams [12,13] (**1**) diols [14,15] (**3** and **4**) and ethers [16] (**5**) which demonstrated *in vitro* HIV-protease activities. The basic structures of the various cage compounds are presented in Figure 1. One cage peptoid [15] also showed promising anti HIV PR activity. The synthesis of the PCU-lactam (**1**) [17-19] and lactone (**2**)^12,^ [18,20] is well established.

**Figure 2:**
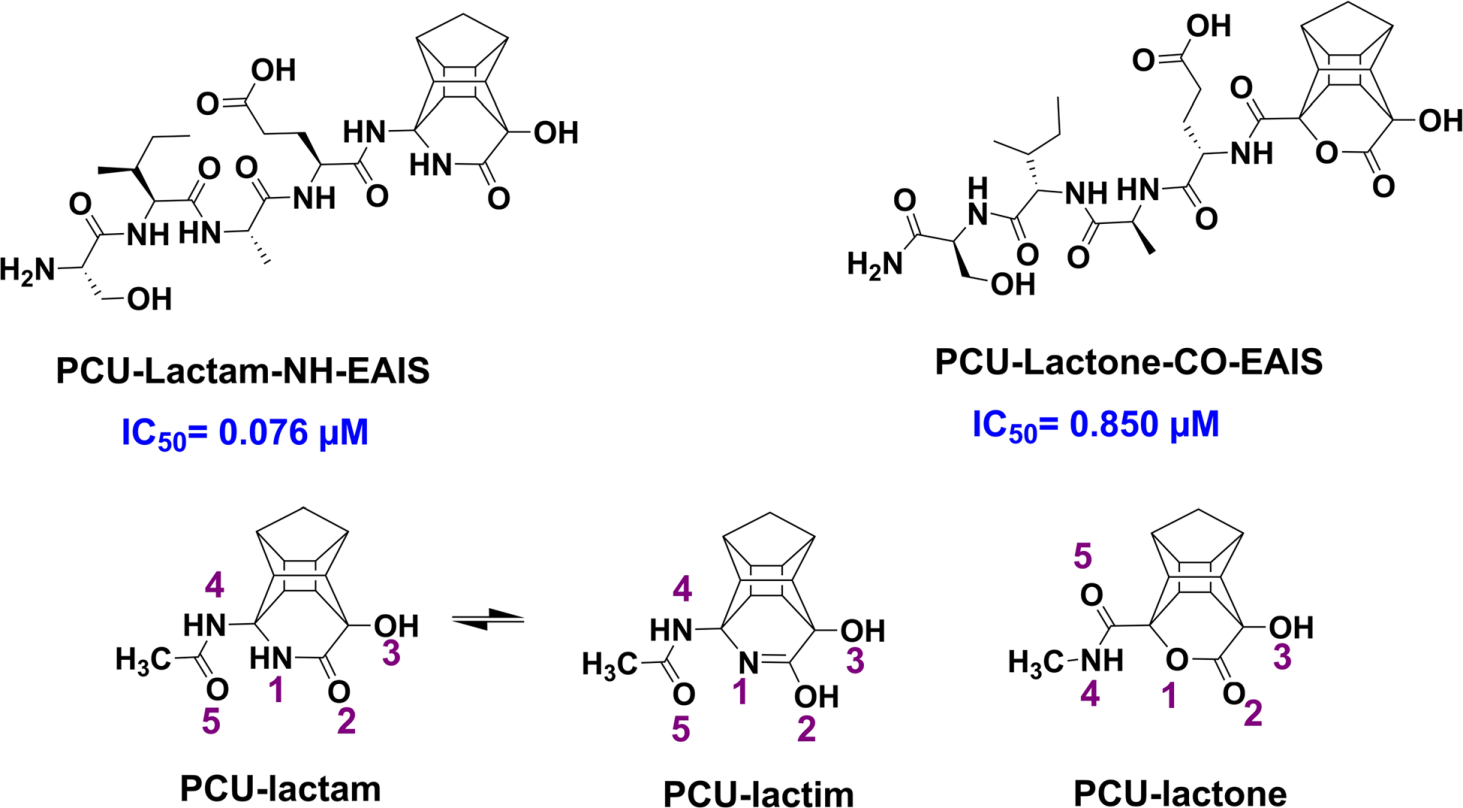
**Structures of PCU-lactam-NH-EAIS and PCU-lactone-CO-EAIS peptide inhibitors and their corresponding PCU-models.** E: Glutamic acid; A: Alanine; I: Isoleucine; S: Serine (Note that the PCU-models consist of two enantiomers. The cage peptides therefore exist as two diastereomers).

In this paper, we followed our PCU-lactam-NH-EAIS peptide (**6**) studies [12,13] based on the synthesis and testing of an analogous novel PCU-lactone-CO-EAIS compound (**7**) (Figure 2). The lactam peptide exhibits an order of magnitude better HIV protease inhibitory activity, in comparison with the lactone analogues. We have recently argued that the activity of PCU-lactam-NH-EAIS peptide stems from its function as a norstatine type transition state analogue [12,13]. Several potential reasons for the discrepancy in HIV PR activities exist. First, the lactam peptide involves C → N amino acid coupling while the lactone peptides consist of N → C coupling; this should induce very different binding energies. Second, the hydrogen bond interaction of the cage lactam group with the protease Asp25/25′ residues may be more advantageous.

The objective of this study is to investigate MD-based binding affinities and electronic structural features of the proposed compounds to obtain a theoretical explanation for the significant difference in the experimental IC_50_ data for lactam and lactone inhibitors [12,13].

Extensive molecular modeling techniques on PCU compounds have been applied in our laboratory [12-16,21-30] and we aimed to use these and alternative quantum chemical approaches [31-39] to gain more insight into the discrepancies of the observed bio-activities.

Structures of PCU-lactam-NH-EAIS, and PCU-lactone-CO-EAIS inhibitors and their corresponding PCU-models are demonstrated in Figure 2.

From a computational standpoint, our strategy in this work is summarized as follows:

First, molecular docking and 10 ns MD calculations have been performed for both the PCU-peptide and model compounds as well as their corresponding diasteromers complexed to South African HIV protease (C-SA) in aqueous solution using both MM-PBSA (explicit water as solvent) and MM-GBSA (implicit solvent) methods. The MM-PBSA method includes the calculation of the molecular mechanics gas phase energies, polar continuum electrostatic solvation energies (by solving the linearized Poisson-Boltzmann equation), and non-polar surface area energies. MM-GBSA is a faster method than MM-PBSA and in this approach the GB model approximates the electrostatic contribution to the free energy of solvation. Both of these methods give efficient, reproducible, and reliable binding free energies of solvation [40-43].

A comparative study to determine the MM-PB(GB)SA Gibbs binding free energies of solvation for the PCU-peptide and model compounds was then applied to compare the binding affinities. This will enable us to determine the cause(s) for the significant difference between the IC_50_ values of PCU-lactam-NH-EAIS in compared with PCU-lactone-CO-EAIS peptides.

Second, we assumed that the considerable difference between experimental IC_50_ values could be due to a higher charge density on the lactam in comparison to lactone. To verify our assumption, further DFT analysis of these models aimed to explore the electronic structural influences of the lactam *versus* the lactone moiety. In this regard, NQR studies [44,45] have emerged as a promising theoretical method to study potential correlations between the bioactivity of compounds and their electronic characteristics. Hence, NQR parameters, namely, asymmetry parameters (η) and quadrupole coupling constants (χ) of nitrogen and oxygen nuclei as well as atomic charges derived from natural population analysis (NPA) were calculated for a PCU-model (Figure 2) derived from PCU-lactam, lactim and lactone-CO-EAIS peptides. The tautomeric form of the lactam *i.e*. lactim [19,20] was studied before.

Finally, to evaluate the natural population analysis (NPA) atomic charges, the electrostatic potentials were calculated and regions rich in electronic charge density were characterized. Polarizability, dipole moment, and Gibbs free energies of solvation (∆G_solv_) values of the title molecules in aqueous solution were also calculated at the same level of theory. Such a theoretical study is useful to address the experimental observations through molecular dynamic and electronic structure features of these inhibitors. The outcome of this work could be helpful for the future design of novel therapeutic agents that inhibit different relevant diseases.

## Methods

### Synthesis of cage lactone peptides

**Scheme 1:**
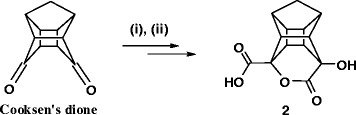
**Synthesis of the cage lactone (i) NaCN, CH3COOH, H2O (ii) 3 M HCl.**

The cage lactone (**2**) [18] was synthesized from Cooksen’s dione [46,47] using a reported procedure utilizing sodium cyanide to generate cyano cage lactam which was converted to cage lactone (**2**) using hydrochloric acid (Scheme 1).

**Scheme 2:**
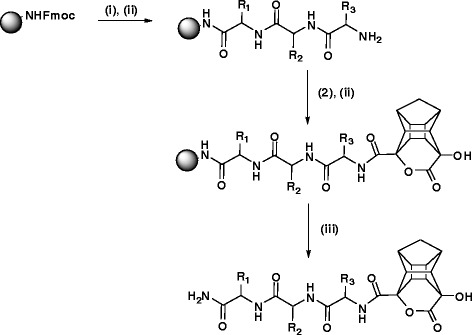
**Synthesis of cage lactone peptides (i) 20:80 piperidine:DMF (ii) HCTU, DIPEA, DMF (ii) 95:5% TFA:DCM.**

The chosen peptide sequence (EAIS, (S)-amino acids were used) is first synthesised on rink resin and the cage lactone is then coupled in the final step. HCTU and DIPEA were used as coupling reagents and piperidine/DMF (2:8) was used to deprotect the Fmoc group from the amino acid. The final cleavage of peptide from the resin was achieved using a mixture of TFA and DCM (95:5% v/v) (Scheme 2).

### Over-expression, extraction and purification of the C-SA protease and *in vitro* HIV-1 protease activity

Over-expression, extraction and purification of the C-SA protease and *in vitro* HIV-1 protease activity were reported in our earlier publications [12,13]. Genetic diversity among HIV-1 subtypes ranges between 25 and 35% at the nucleotide level and variation within subtypes vary from 15 to 20% [48,49]. Thus, both the high mutation rate within the HIV PR and the genetic variation of HIV is an ongoing challenge toward finding a potent protease inhibitor (PI) with reasonable inhibition of various HIV subtypes [50].

The HIV-1 subtype C PR that predominates in South Africa (C-SA PR) is defined as the consensus subtype C PR [51]. This C-SA PR contains eight polymorphic sites (T12S, I15V, L19I, M36I, R41K, H69K, L89M, and I93L) in relation to the wild-type subtype B HIV-1 PR [52]. Multiple sequence alignment of the C-SA PR, subtype B PR, subtype B-MDR PR, and subtype C-2R8N PR confirmed that subtype C of South African HIV-1 protease is structurally very close to the rest of the HIV-1 proteases [53].

The procedures followed were the same as reported before ^12,13^.

### Molecular dynamic simulation

The geometry optimizations of the considered ligands were performed using the GAUSSIAN03 package [54] at the B3LYP theoretical level and with the 6-311G(d,p) basis set. Afterwards, the partial atomic charges were calculated using the restricted electrostatic potential (RESP) [55,56] method using HF/6-31G(d). The RESP charges were generated based on the calculated electrostatic potential using antechamber implemented in the AMBER 12 package [57,58]. The force field parameters for the ligands were described by General Amber Force Field (GAFF) [59]. The standard AMBER ff99SB force field [60,61] for bio-organic systems was used to describe the protein parameters.

Starting structures for MD simulations were obtained by molecular docking of PCU-models and PCU-EAIS peptide inhibitors as well as their corresponding diastereomers inside the active pocket of HIV PR using Autodock4 program [62]. In order to make sure that all the structures have the same orientation inside the active pocket of HIV PR, all docked structures were aligned using Pymol [63] program. Protons were added to the protein in accordance with pKa calculations using PropKa 3.1 server [64]. The physiological pH conditions and the correct protonation state of ionizable groups in the CSA HIV PR enzyme was considered for molecular dynamic simulations. In this context, one of the aspartates (Asp25) of the catalytic site of the the CSA HIV PR exhibits an increased pKa value of 5.2 in the inhibitor-bound protease [65] while no increased pK_a_ was observed for the free form of the protease (pKa. 4.5) [66]. We have previously reported [14] that different protonation states do not have an adversed effect on the results.

The Sander module implemented in AMBER 12 [57] together with AMBER ff99SB force field [60,61] were used to perform all MD simulations as well as for the minimizations and equilibration protocols. Each system was solvated in a truncated cubic TIP3P water [67] box with 10 Å distance around the complex. Minimizations were carried out in a constant volume (NVT ensemble) by 5000 cycles of steepest descent minimization followed by 5000 cycles of conjugated gradient minimization under harmonic restraints with force constant 500 kcal · mol^−1^ · Å^−2^ to all solute atoms. Then, the minimization was followed in a constant volume by 10000 cycles of steepest descent minimization followed by 10000 cycles of conjugated gradient without any harmonic restraints for all atoms of the system. Then, each system was gently annealed from 0 K up to 300 K for 70 ps with an isobaric ensemble. Finally, the 10 ns canonical ensemble (NVT) MD simulations were applied without any restraints. The temperature was regulated at 300 K using the Langevin thermostat [68]. For all MD simulations, 2 fs time step and 12 Å non-bonded cutoff were used. The particle mesh Ewald method [69] was used to treat long-range electrostatics, and bond lengths involving bonds to hydrogen atoms were constrained with the SHAKE algorithm [70]. The root mean square deviation (RMSD) of the average structures over the MD trajectory between a defined starting point of the simulation and all succeeding frames were also calculated.

Since the chances of the inhibitor/complex being trapped as a local minimum structure is quite high, an iterative process was followed where the inhibitor peptide backbone from the lowest energy complex [say PCU-lactam-NH-EAIS(a)] for each of the diastereomeric peptides (see Tables 1 and 2) was imposed on the related analogue [i.e. PCU-lactone-NH-EAIS(a)], for a subsequent MD run. The positions of the pairs of diastereomers (lactam *versus* lactone) with respect to the active site (particularly with respect to the catalytic Asp25/25′ residues) during the MD were monitored to ensure that the two inhibitors maintained very similar positions during the MD simulations. Several different docked starting structures were used in order to ensure that the lowest possible energy inhibitor/complexes were obtained. This procedure also enabled us to draw meaningful comparisons between the binding energies of the respective lactam/lactone-peptide diastereomers.

**Table 1:** **Binding free energies of solvation and its components for the PCU-based ligands complexed with the HIV protease in in kcal/mol (the 3D structures of these compounds are provided with the supporting information)**

**Ligand** ^**a**^	**ΔE**_**Ele**_	**ΔE**_**VDW**_	**ΔE**_**Sol**_ **(PB)**	**ΔE**_**Sol**_ **(GB)**	**ΔG**_**Bind**_ **(PBSA)** ^**b**^	**ΔG**_**Bind**_ **(GBSA)** ^**b**^
**PCU-lactam-NH-EAIS(a)**	−22.45	−69.26	55.49	48.12	−45.36	−52.63
**PCU-lactone-NH-EAIS(a)** ^**c**^	−22.36	−62.33	49.97	48.90	−41.28	−43.39
**PCU-lactam-NH-EAIS(b)**	−22.25	−63.76	55.16	46.05	−40.31	−47.06
**PCU-lactone-NH-EAIS(b)** ^**c**^	−22.67	−59.18	50.16	45.94	−39.36	−42.95
**PCU-lactam-CO-EAIS(a)** ^**c**^	−23.82	−64.47	47.35	43.28	−45.89	−49.18
**PCU-lactone-CO-EAIS(a)**	−11.47	−57.01	39.23	34.16	−41.77	−46.36
**PCU- lactam-CO-EAIS(b)** ^**c**^	−23.82	−64.47	46.58	43.28	−45.89	−49.18
**PCU-lactone-CO-EAIS(b)**	−20.95	−62.08	50.62	46.85	−41.35	−44.01
**PCU-lactam(a)**	−1.48	−34.13	12.73	12.39	−29.52	−27.63
**PCU-lactone(a)**	−2.51	−25.61	14.05	13.34	−28.19	−17.37
**PCU-lactam(b)**	−1.88	−30.14	14.94	14.99	−29.69	−19.99
**PCU-lactone(b)**	−5.30	−27.26	17.60	17.53	−23.39	−17.92

^a^(**a**) and (**b**) refer to the enantiomers of the models and the diasteromeric peptides, respectively.

^b^Note that the total free energy of solvation values in AMBER were used and not the Quasi-harmonic Entropy Approximation energies.

^c^Theoretically designed PCU-lactone-peptide inhibitors analogous to the previously synthesised PCU-lactam-peptides.

**Table 2:** **Binding free energies of solvation and its components using normal mode analysis for the PCU-based ligands complexed with the HIV protease in kcal/mol**

**Ligand** ^**a**^	**ΔE**_**Ele**_	**ΔE**_**VDW**_	**ΔE**_**Sol**_ **(PB)**	**ΔE**_**Sol**_ **(GB)**	**ΔG**_**Bind**_ **(PBSA)**	**ΔG**_**Bind**_ **(GBSA)**
**PCU-lactam-NH-EAIS(a)**	−20.38	−67.15	45.78	44.53	−46.91 (-20.10)^b^	−54.76 (-22.37)^b^
**PCU-lactone-NH-EAIS(a)** ^**c**^	−26.90	−64.81	56.64	54.19	−42.62 (-17.09)^b^	−45.37 (-19.62)^b^

^a^(**a**) refers to one of the enantiomers of the models and the diasteromeric peptides, respectively.

^b^ΔG binding values using normal mode entropy approximation.

^c^Theoretically designed PCU-lactone-peptide inhibitors with the same side chain order than the previously synthesised PCU-lactam-peptides.

In order to obtain a better theoretical understanding about the difference characteristics between the lactam/lactone-peptides, two pairs of theoretical cage peptides were studied, namely PCU-lactone-NH-EAIS(**a** and **b**) and PCU-lactam-CO-EAIS(**a** and **b**). Due to the current lack of synthetic procedures for the required lactam/lactone starting structures, the synthesis of these peptides are not feasible.

### MM-PB (GB) SA binding free energy calculations

MM-PB (GB) SA (Molecular Mechanics-Poisson-Boltzmann or Generalized Born solvent-accessible Surface Area) methods provide an effective computational tool in the analysis of biomolecular interaction [71-73]. This approach is based on the calculation of the average free energies of solvation (ΔG_bind_) between a target protein and a set of ligands over the trajectory of molecular dynamics (MD) simulation. In this method [74,75], the ΔG_bind_ between a ligand (L) and a receptor (R) to form a complex (RL) is defined as:

1 $$ \Delta {\mathrm{G}}_{\mathrm{Bind}}=\Delta \mathrm{H}\ \hbox{--}\ \mathrm{T}\mathrm{D}\mathrm{S} \mbox{\fontencoding{T1}\fontfamily{cmr}\selectfont\char"14} \Delta {\mathrm{E}}_{\mathrm{MM}}+\Delta {\mathrm{G}}_{\mathrm{sol}}\hbox{--}\ \mathrm{T}\mathrm{D}\mathrm{S} $$

2 $$ \Delta {\mathrm{E}}_{\mathrm{MM}}=\Delta {\mathrm{E}}_{\mathrm{Int}}+\Delta {\mathrm{E}}_{\mathrm{E}\mathrm{le}}+\mathrm{D}{\mathrm{G}}_{\mathrm{VDW}} $$

3 $$ \Delta {\mathrm{E}}_{\mathrm{Sol}}=\Delta {\mathrm{G}}_{\mathrm{PB}/\mathrm{GB}}+\Delta {\mathrm{G}}_{\mathrm{SA}} $$

Where ΔE_MM_, ΔG_Sol_ and -TΔS are the changes of the gas phase MM energy, the free energy of solvation, and the conformational entropy upon binding, respectively. The ΔE_MM_ term includes ΔE_Int_ (bond, angle, and dihedral energies), ΔE_Ele_ (electrostatic), and ΔE_VDW_ (van der Waals) energies. The ΔG_Sol_ is the solvation binding free energy as the sum of polar (electrostatic solvation energy) and nonpolar free energies of solvation (Δ*G*_PB/GB_, and the non-electrostatic solvation component, ΔG_SA_). The polar contribution is calculated using either the GB or PB model, while the nonpolar energy is estimated by solvent accessible surface area (SASA) [76,77].

### DFT study

Gas phase calculations were performed by using GAUSSIAN09 [78] density functional theory (DFT) with the B3LYP functional [79,80]. The 6-311G(d,p) basis set was used for both the geometry optimization and electronic structure calculations. This basis set includes diffuse functions on hydrogen and the heavy atoms and is a suitable option for polar molecules with electron lone-pairs [81]. B3LYP non-local hybrid exchange correlation functional that includes a mixture of Hartree–Fock exchange with DFT exchange–correlation is known as the commonplace substitute approximation. LYP supports the full correlation energy and not only a correction to local spin density approximation (LSDA), its overall performance is sufficiently good for organic molecules [82]. Harmonic vibrational frequencies were also calculated to confirm that all structures were minima on the potential energy surface and to calculate the zero-point vibrational energy (ZPVE) and the Gibbs free energy. Various atomic and molecular electronic structure quantities were calculated from optimized structures. For the calculation of atomic charges, natural bond orbital analysis (NBO) [83-85] was performed as well as the natural population analysis (NPA) as an optimal wave function-based method. To accurately analyze the charge distribution around these atoms, the electrostatic potential energy values were calculated and mapped over an isodensity surface corresponding to 0.002 a.u. This electrostatic potential surface includes the van der Waals volumes of the individual atoms in the molecule and is thus a good representation of the reactive regions around the molecules [86,87].

Moreover, the NQR spectral quantities including asymmetry parameter (η) and quadrupole coupling constant (χ) of nitrogen and oxygen atoms, shine useful insight on biological effectiveness of drug-like compounds [44]. These two quantities can be derived from the electric field gradient tensor using the following equations:

4 $$ \upchi =\frac{{\mathrm{e}}^2\mathrm{Q}\left\langle \mathrm{q}\mathrm{z}\mathrm{z}\right\rangle }{\mathrm{h}} $$

5 $$ \upeta =\frac{\left\langle \mathrm{q}\mathrm{z}\mathrm{z}\right\rangle -\left\langle \mathrm{q}\mathrm{y}\mathrm{y}\right\rangle }{\left\langle \mathrm{q}\mathrm{z}\mathrm{z}\right\rangle } $$

Where e < q_xz_>, e < q_yy_ > and e < q_zz_ > are the principal components of the effective electric field gradient tensor defined such that │ < q_zz_│ < q_yy_ > │ < q_xx_> > │and Q is the nuclear electric quadrupole moment [88]. The electric quadrupole moments (Q) of ^17^O and ^14^ N atoms were taken as 25.58× 10^−27^ cm^2^ and 20.44 × 10^−27^ cm^2^, respectively [89].

We calculated the asymmetry parameters (η) and quadrupole coupling constants (χ) of nitrogen and oxygen atoms involved in the cage region of PCU-lactam, its tautomer lactim [19,20] and PCU-lactone models in vacuum and in solvent media (water, DMSO and dioxane). The solvation effect (aqueous solution) on the Gibbs free energy of solvation (ΔG_sol_) were also evaluated utilizing the self-consistent reaction field (SCRF) keyword [90] with the solvation model on density (SMD) [91]. The SMD model computes the electrostatic interaction based on the integral-equation-formalism polarizable continuum model (IEF-PCM) and complements the description of the solute-solvent interaction by adding the missing non-electrostatic term to make accurate predictions of ΔG_sol_. The latter were calculated in vacuum/gas phase and in solvent (water) for the titled systems by taking the difference of the obtained ΔG_sol_ values in both gas and aqueous media.

## Results and discussion

### Inhibitory activity of anti-HIV PR compounds

**Figure 3:**
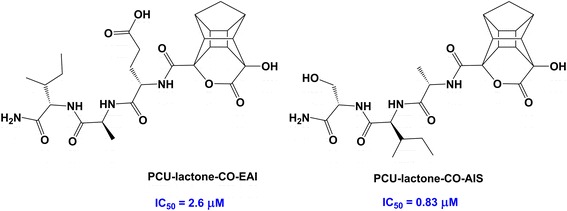
**The structures of PCU-lactone-CO-peptides.**

The activity data of HIV PR inhibitors are presented in Table 3. Peptides of the cage lactone furnished significant HIV PR activities. However, the PCU-lactone-CO-EAIS peptide (Figure 2) exhibited an IC_50_ value of ~0.85 μM, which was eleven times weaker than the corresponding cage lactam peptide (IC_50_ ~ 0.078 μM). The shorter PCU-lactone-CO-EAI peptide (Figure 3) demonstrated poor activities (IC_50_ > 2.6 μM) while the PCU-lactone-CO-AIS peptide was a better inhibitor (IC_50_ ~ 0.83 μM) (Figure 3).

**Table 3:** **Wild type CSA HIV-1 protease inhibition activities of PCU-lactam-NH- and lactone-CO-peptides**

	**Peptide**	**IC**_**50**_ with lactam-NH- (μM)*	**IC**_**50**_ with lactone-CO- (μM)*
**Mixture of both diastereomers**	Glu-Ala-Ile-Ser	0.078 ± 0.0035	0.85 ± 0.18

*IC_50_ = 50% inhibition constant reported as the average of three experiments. Standard deviations (SD) for all compounds were ≤ 2% of the reported IC_50_ values. Peptides were isolated as a mixture of diastereomers. Atazanavir (IC_50_ = 0.004 ± 0.00071 μM) and Lopinavir (IC_50_ = 0.025 ± 0.0014 μM) were used as standards for the testing.

### MM-PB (GB) SA binding free energy analysis

**Figure 4:**
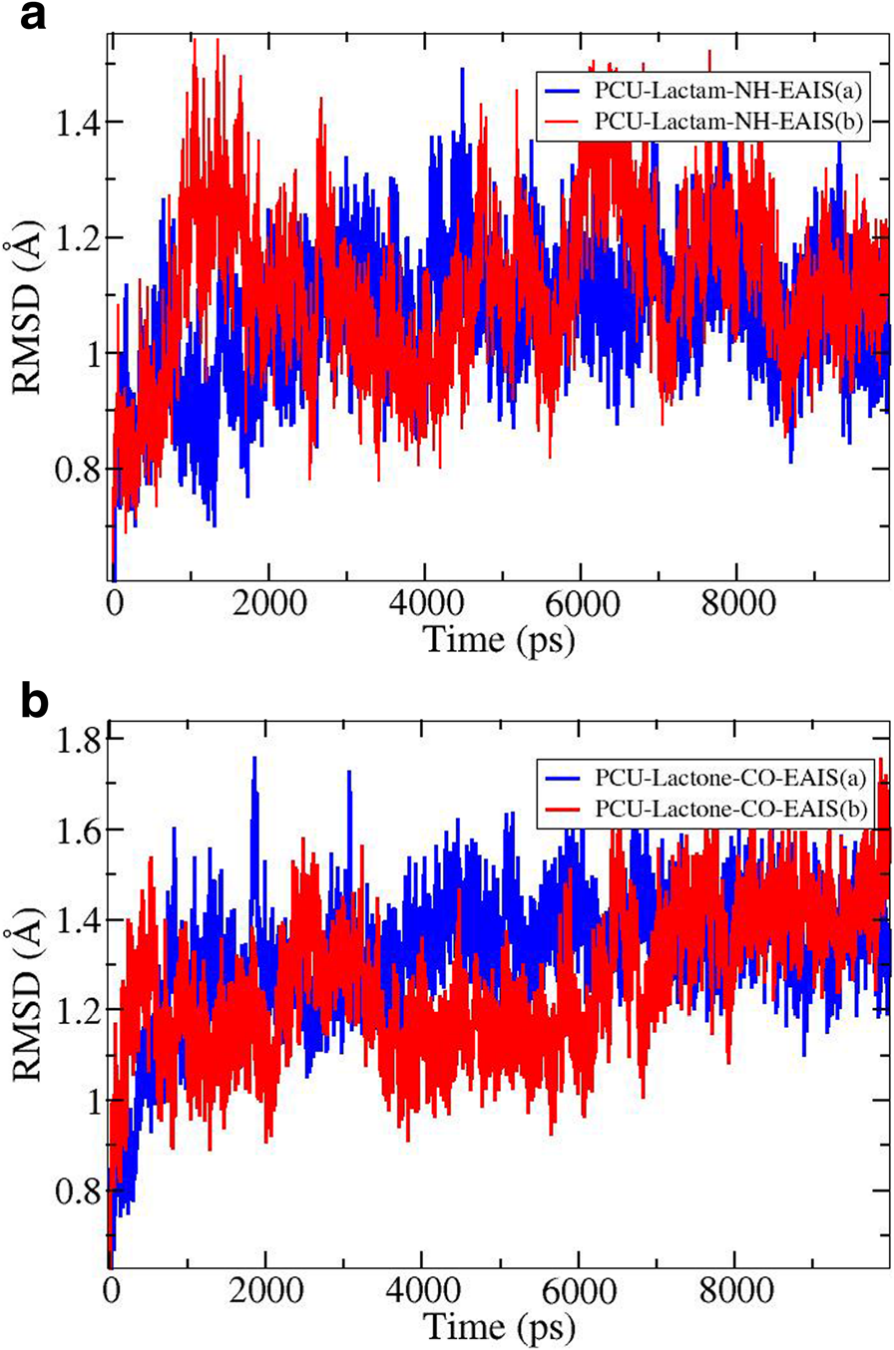
**Analysis of the RMSD of energy minimized (1) PCU-lactam-NH-EAIS (a,b) and (2) PCU-lacton-NH-EAIS (a,b) obtained over the course of 10 ns MD trajectories.** a and b = diastereomers [MD in explicit solvent model].

MM-PB (GB) SA binding free energy method was employed to estimate the average free energies of solvation of the titled complexes obtained over 10 ns MD simulations. The dynamics and stability of the considered complexes were also investigated based on the root mean square deviation (RMSD) graphs. The RMSD graphs of PCU-lactam-NH-EAIS (a,b) and its corresponding lactone, *i.e*., PCU-lactone-NH-EAIS (a,b) structures over 10 ns MD trajectories are depicted in Figure 4.

According to the plotted RMSD graphs in Figure 4, all the considered PCU-EAIS peptides maintained the same level of stability with an RMSD of 0.80 Å during the entire time of the 10 ns MD simulation.

In order to calculate the theoretical binding energies, we used our MD simulations at 10 ps intervals, as suggested by Stoica and co-workers [92]. The free energies of solvation for all selected complexes averaged over the trajectories of the explicit water 10 ns MD simulations and are listed in Tables 1 and 2. Note that ΔG_bind_ for both MM-PB(GB)SA methods and its components including polar electrostatic (ΔE_Ele_), van der Waals (ΔE_VDW_) and PB or GB solvation energies [ΔE_Sol_(GB) and ΔE_Sol_(PB)] are defined in equations 1, 2 and 3.

The first important observation is that for all cases, the PCU-lactam-peptides diastereomers showed better binding energies (more negative ΔG_bind_) than the corresponding lactone-peptide diastereomers. The theoretical results [both ΔG_Bind_(PBSA) and ΔG_Bind_(GBSA)] for Lactam-NH-EAIS(a) peptide showed better binding affinity against CSA-HIV PR in comparison to PCU-lactone-CO-EAIS(a) peptide inhibitors. On the other hand, the case for (b) has slightly higher binding energy with ΔG_Bind_(PBSA) for the lactam peptide, while with ΔG_Bind_(GBSA) the same pattern as before was observed (better/lower binding energy for the lactam peptide). These results largely support the lower experimental IC_50_ values for PCU-lactam-NH-EAIS inhibitor (IC_50_ = 0.076 μm) than the PCU-lactone-CO-EAIS compound (IC_50_ = 0.850 μm).

The same trend is observed for the calculated binding energies of the isolated lactam/lactone model compounds. The lactam (both enantiomers) give better binding energies than the corresponding lactone. It therefore appears likely that the source of better binding energy for the cage peptides originates from the PCU skeleton.

Since the experimental IC_50_ values for the peptides were measured for a diastereomeric mixture [resulting from the two cage enantiomers attached to the (S)-peptide] it makes sense to look at the average of the calculated binding free energies of the PCU model compounds as well. In the case of both PB and GB solvation models the same order for binding free energy values was observed for the PCU models (top four models in Tables 1 and 2): Lactam-PCU < Lactone PCU (*i.e.* PCU-lactam models (either **a** or **b**) exhibit stronger ΔG_bind_ in comparison to the PCU-lactone). It is important to note that for both cases (PCU-model and peptide) that the PCU-lactone consistently gave the weakest binding energies.

Amongst the component of binding free energies, the van der Waals (VDW) interactions between the ligands and the HIV protease showed the largest contribution to the ΔG_bind_. This is the case for both MM-PBSA and MM-GBSA methods. However, the mentioned difference was more evident in MM-GBSA data. Two cases for the electronic energy contributions are worth pointing out. Lower ΔE_Ele_ value (−11.47 kcal/mol - smaller difference) was reported for the PCU-lactone-CO-EAIS(a) in comparison to the PCU-lactam-NH-EAIS(a) peptide (−22.36 kcal/mol). Lower ΔE_Ele_ value of this compound is attributed to the potential electronic interactions (Coulombic, dipole-dipole interactions, hydrogen bonds *etc*.). We have previously demonstrated [12,13] that the hydroxyl group (−O_3_H, see Figure 2) of the PCU-lactam is very important for HIV PR activity since it is presumed to interact with the two catalytic Asp25/25′ residues of HIV PR. Analysis of this distance between the PCU hydroxyl group (−O_3_H) and Asp revealed a weaker interaction for the PCU-lactam-NH-EAIS (average of about 13 Å) than for the corresponding PCU-lactone peptide (average of about 6.5 Å, see Additional file 1). This can potentially explain the lower ΔE_Ele_ value for the PCU-lactone-CO-EAIS (a).

We calculated the entropic contribution using normal mode analysis for the PCU-lactam-NH-EAIS(a) and PCU-lactone-NH-EAIS(a) (Table 2).

The binding free energy has slightly improved, but the same trend was observed.

A more detailed study will be reported later to address the potential segment of the PCU-based compounds causing this change in their binding affinities.

### Electronic structural analysis

**Figure 5:**
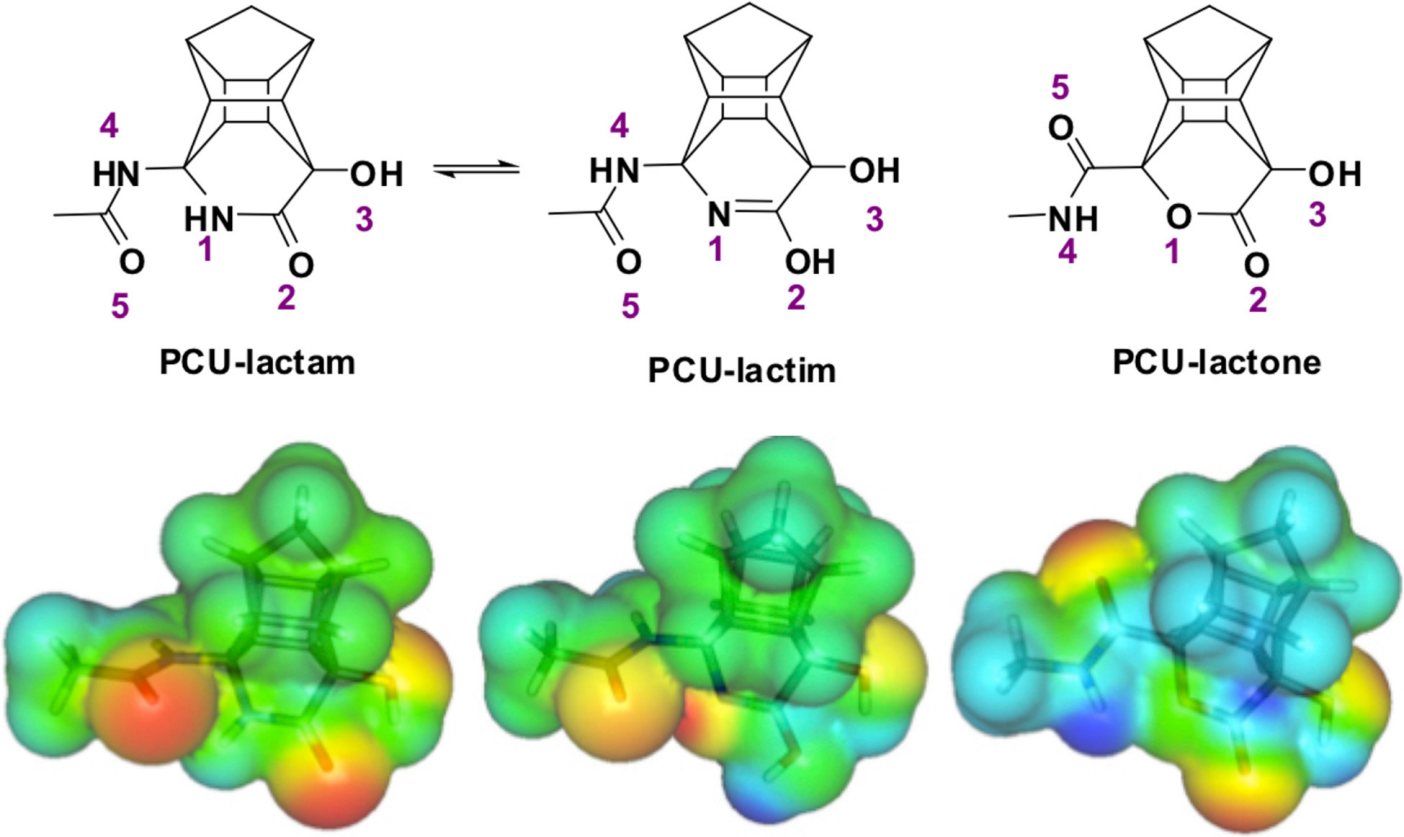
**The electrostatic potential map [B3LYP/6-311G(d,p)] for PCU models in vacuum.** (red = electron rich and blue = electron defficient).

In this section, we considered the PCU-lactam, lactim and lactone models shown in Figure 5. The two reported sets of ^1^H NMR signals in various solvents suggested the lactim–lactam tautomeric equilibrium exists [19,20]. In (CD_3_)_2_SO the major tautomer is the lactam form, while for dioxane the lactim is dominant. It is therefore most likely that the lactam form is the active form in aqueous solution (also used for the IC_50_ experiments). The conformation for the amide side chain with the lowest energy was determined. In both cases this conformation involved intramolecular hydrogen bonding (O_5_ - - HN_1_ for the lactam and N_4_H - - O_1_ for the lactone).

First, we have focused on the natural atomic charges obtained from NBO analysis and then we assessed the electrostatic potential map produced with the Molekel [93] program. NBO analysis calculates atomic charges, by summing occupancy of natural atomic orbitals (NAOs). These charge values for nitrogen and oxygen nuclei of the considered models are presented in Table 4.

**Table 4:** **Natural atomic charges (a.u.) on nitrogen and oxygen nuclei of PCU-lactam, PCU-lactim and PCU-lactone models obtained from NBO analysis [B3LYP/6-311G(d,p)]**

**Atom**	**Lactam**	**Lactim**	**Lactone**
**N**_**1**_ **/O**_**1**_	−0.65213	−0.56236	−0.58068
**O**_**2**_	−0.63617	−0.69693	−0.59012
**O**_**3**_	−0.74727	−0.73899	−0.73981
**N**_**4**_	−0.65298	−0.65717	−0.60787
**O**_**5**_	−0.63987	−0.62519	−0.65063

According to the reported data in Table 4, larger negative atomic charges were observed for most of the hetero-atoms (N_1_, O_2_, O_3_ and N_4_) of PCU-lactam model in comparison to the corresponding atoms in the lactone case. The less negative atomic charge of O_5_ for PCU-lactam (−0.63987 a.u) than the corresponding value for the PCU-lactone (−0.65063 a.u) can be explained due to the intramolecular hydrogen bonding interaction in the lactam compound.

Analysis of the charge properties mapped onto the optimized structure will assist with the characterization of how the electronic properties are related to chemical activity [86]. In addition, electrostatic potential map (ESP) exhibits the charge distribution of a molecule based on the properties of the nucleus and nature of electrostatic potential energy. The molecular ESPs of these compounds are illustrated in Figure 5. This assists one to visualize different charged regions of these molecules.

**Figure 6:**
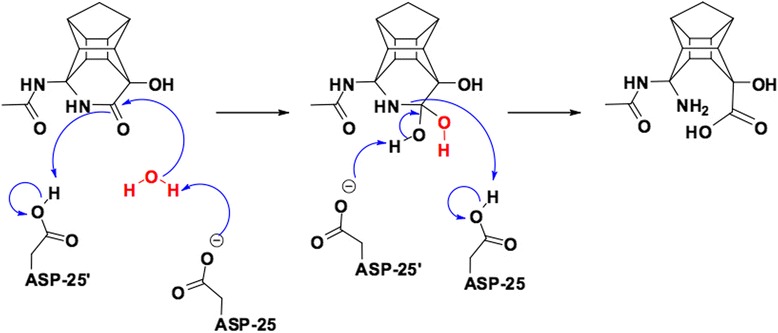
**Potential reaction of PCU-lactam-NH-AIS peptide inhibitors with HIV protease based on the general HIV PR mechanism** [94]**.**

The lactam structure which is supposed to interact with the two aspartic acid residues in the HIV PR (Figure 6, based on general mechanism) [94] is clearly more electron rich in the area (O_2_ and O_5_ atoms) (Figure 5). The corresponding regions for the lactone (O_2_ and N_4_H) appeared less electron rich for O_2_ and electron difficient for N_4_H. With the C = O_2_ group of the PCU-lactam model, clearly more polarized than the corresponding carbonyl of the lactone. To recognize reactive domains on the compound’s surface in solution, the calculation of ESP charges was also performed in water. According to the obtained ESP values (data are provided in Additional file 1), it is evident that the polarization of these compounds in aqueous medium increase. Again, the same trend is observed. It can be envisaged from the mechanism of HIV-protease that interaction between the aspartic acid groups and the lactam may be more pronounced. This is potentially a reason for better activity (lower IC_50_) for PCU-lactam inhibitors in comparison to the lactone series.

The electronic charge distribution around the nitrogen and oxygen nuclei was further investigated through the calculation of asymmetry parameters (η) and quadrupole coupling constants (χ) generally known as NQR parameters. The calculated parameters for the ^14^N and ^17^O atoms of PCU-lactam, lactim and PCU-lactone models in vacuo, water, DMSO, and dioxane are reported in Table 5. These values were obtained from the electric field gradient (EFG) tensor calculations and characterized the local electron density distribution around these atoms. Indeed, the interaction of the nuclear quadrupole moment (Q) with the EFG identifies the degree of the double bond character of chemical bonds in which the quadrupole nuclei is involved [88]. The relationship between asymmetry parameters (η) and quadrupole coupling constants (χ) for oxygen atoms (including carbonyl and alcohol oxygens) was studied [95]. It was reported that the relationship is ordered, but not necessarily linear.

**Table 5:** **Asymmetry parameter (η) and the quadrupole coupling constant (χ) (MHz) of the** ^**14**^ **N and** ^**17**^ **O atoms for the PCU-lactam, PCU-lactim and PCU-lactone models [B3LYP/6-311G(d,p)] in different solvent media**

**Lactam**								
**Atom**	**Vacuum**		**Water**		**DMSO**		**Dioxane**	
	**η**	**χ (MHz)**	**η**	**χ (MHz)**	**η**	**χ (MHz)**	**η**	**χ (MHz)**
**N**_**1**_	0.1593	4.553	0.1169	4.230	0.1110	4.390	0.1403	4.473
**O**_**2**_	0.1148	8.777	0.4479	8.284	0.3289	8.335	0.1960	8.607
**O**_**3**_	0.9180	11.391	0.9060	11.231	0.8987	11.370	0.9116	11.377
**N**_**4**_	0.1051	4.837	0.2232	4.417	0.2015	4.613	0.1400	4.732
**O**_**5**_	0.1569	9.147	0.3469	9.064	0.1880	9.306	0.1945	9.111
**Lactim**								
**Atom**	**Vacuum**		**Water**		**DMSO**		**Dioxane**	
	**η**	**χ (MHz)**	**η**	**χ (MHz)**	**η**	**χ (MHz)**	**η**	**χ (MHz)**
**N**_**1**_	0.3252	3.578	0.4653	3.434	0.9999	3.430	0.3681	3.421
**O**_**2**_	0.9131	11.752	0.9492	9.817	0.7041	9.937	0.7447	9.960
**O**_**3**_	0.9131	11.752	0.9070	11.397	0.8833	11.751	0.8966	11.769
**N**_**4**_	0.0714	4.884	0.1559	4.253	0.1372	4.509	0.0897	4.749
**O**_**5**_	0.1265	9.267	0.2889	9.408	0.1208	9.698	0.1271	9.918
**Lactone**								
**Atom**	**Vacuum**		**Water**		**DMSO**		**Dioxane**	
	**η**	**χ (MHz)**	**η**	**χ (MHz)**	**η**	**χ (MHz)**	**η**	**χ (MHz)**
**O**_**1**_	0.5128	9.763	0.4787	9.623	0.4827	9.647	0.4961	9.702
**O**_**2**_	0.1356	9.006	0.0348	8.860	0.0509	8.883	0.0934	8.949
**O**_**3**_	0.9212	11.611	0.9172	11.436	0.9100	11.594	0.9176	11.598
**N**_**4**_	0.2087	4.456	0.2584	3.954	0.2277	4.225	0.2182	4.336
**O**_**5**_	0.3060	9.821	0.2367	9.512	0.0867	9.781	0.0627	9.790
**Lactam**								
**Atom**	**Vacuum**		**Water**		**DMSO**		**Dioxane**	
	**η**	**χ (MHz)**	**η**	**χ (MHz)**	**η**	**χ (MHz)**	**η**	**χ (MHz)**
**N**_**1**_	0.1593	4.553	0.1169	4.230	0.1110	4.390	0.1403	4.473
**O**_**2**_	0.1148	8.777	0.4479	8.284	0.3289	8.335	0.1960	8.607
**O**_**3**_	0.9180	11.391	0.9060	11.231	0.8987	11.370	0.9116	11.377
**N**_**4**_	0.1051	4.837	0.2232	4.417	0.2015	4.613	0.1400	4.732
**O**_**5**_	0.0360	9.821	0.2367	9.512	0.0867	9.781	0.0627	9.790
**Lactim**								
**Atom**	**Vacuum**		**Water**		**DMSO**		**Dioxane**	
	**η**	**χ (MHz)**	**η**	**χ (MHz)**	**η**	**χ (MHz)**	**η**	**χ (MHz)**
**N**_**1**_	0.3252	3.578	0.4653	3.434	0.9999	3.430	0.3681	3.421
**O**_**2**_	0.9131	11.752	0.9492	9.817	0.7041	9.937	0.7447	9.960
**O**_**3**_	0.9131	11.752	0.9070	11.397	0.8833	11.751	0.8966	11.769
**N**_**4**_	0.0714	4.884	0.1559	4.253	0.1372	4.509	0.0897	4.749
**O**_**5**_	0.0265	9.990	0.2889	9.408	0.1208	9.698	0.0271	9.918
**Lactone**								
**Atom**	**Vacuum**		**Water**		**DMSO**		**Dioxane**	
	**η**	**χ (MHz)**	**η**	**χ (MHz)**	**η**	**χ (MHz)**	**η**	**χ (MHz)**
**O**_**1**_	0.5128	9.763	0.4787	9.623	0.4827	9.647	0.4961	9.702
**O**_**2**_	0.1356	9.006	0.0348	8.860	0.0509	8.883	0.0934	8.949
**O**_**3**_	0.9212	11.611	0.9172	11.436	0.9100	11.594	0.9176	11.598
**N**_**4**_	0.2087	4.456	0.2584	3.954	0.2277	4.225	0.2182	4.336
**O**_**5**_	0.1569	9.147	0.3469	9.064	0.1880	9.306	0.1945	9.111

The departure of the EFG tensor from the axial symmetry is characterized by the parameter η. The larger the value, the smaller the deviation from axial symmetry. This deviation can be caused by electronic influences such as by hydrogen bonding or packing forces in crystals.

A higher value of the asymmetric parameter (η), around 0.9 was obtained for the oxygen atoms of the lactim hydroxyl group (O_2_) than that of the lactam carbonyl oxygen atom (0.1). It is notable that larger values for η were observed in more polar solvents, as expected.

From the comparison of the measured IC_50_ for the synthesized peptides with the obtained ΔG_Bind_(PB/GB-SA) values it was argued that the source of stronger binding affinity for the cage peptides originates from the PCU skeleton. In order to verify our assumption, it serves best to focus on the quadrupole coupling constant data (**χ**) of the cage (PCU) part of the molecule, which is also expected to interact with the aspartase segments of the PR (Figure 6). Our previous results have shown that O_2_ and O_3_ atoms are crucial in this regard for the PCU-lactam-NH-EAIS peptide inhibitors [12,13]. According to the χ values of the hetero-atoms, it is clear that in each of the four cases (vacuum, water, DMSO and dioxane) the χ values for these atoms (O_2_ and O_3_) were smaller for the PCU-lactam than for the lactone model. Smaller χ values implied that the quadrupole charge on these atoms were more delocalised. This will enable a stronger hydrogen bond interaction of these atoms (O_2_ and O_3_) with the Asp25/Asp25′ groups of the PR. The same observation holded for O_3_ of the lactim compound. As was the case with the asymmetry parameter, the quadrupole constant of O_2_ atom for the lactim also reflects the change from carbonyl oxygen to the oxygen atom in hydroxyl group.

The oxygen atom (O_5_) involved in the lactam side chain carbonyl exhibited less charge density (lower χ values) *versus* the corresponding lactone oxygen atom. Since the lactam side chain carbonyl (O_5_) is in fact involved in intramolecular hydrogen bonding with its NH group (N_1_H), it should be more delocalized resulting in a smaller χ value. This observation is in good agreement with the reported natural atomic charges on the oxygen nuclei of PCU-lactam and lactim (Table 4).

**Figure 7:**
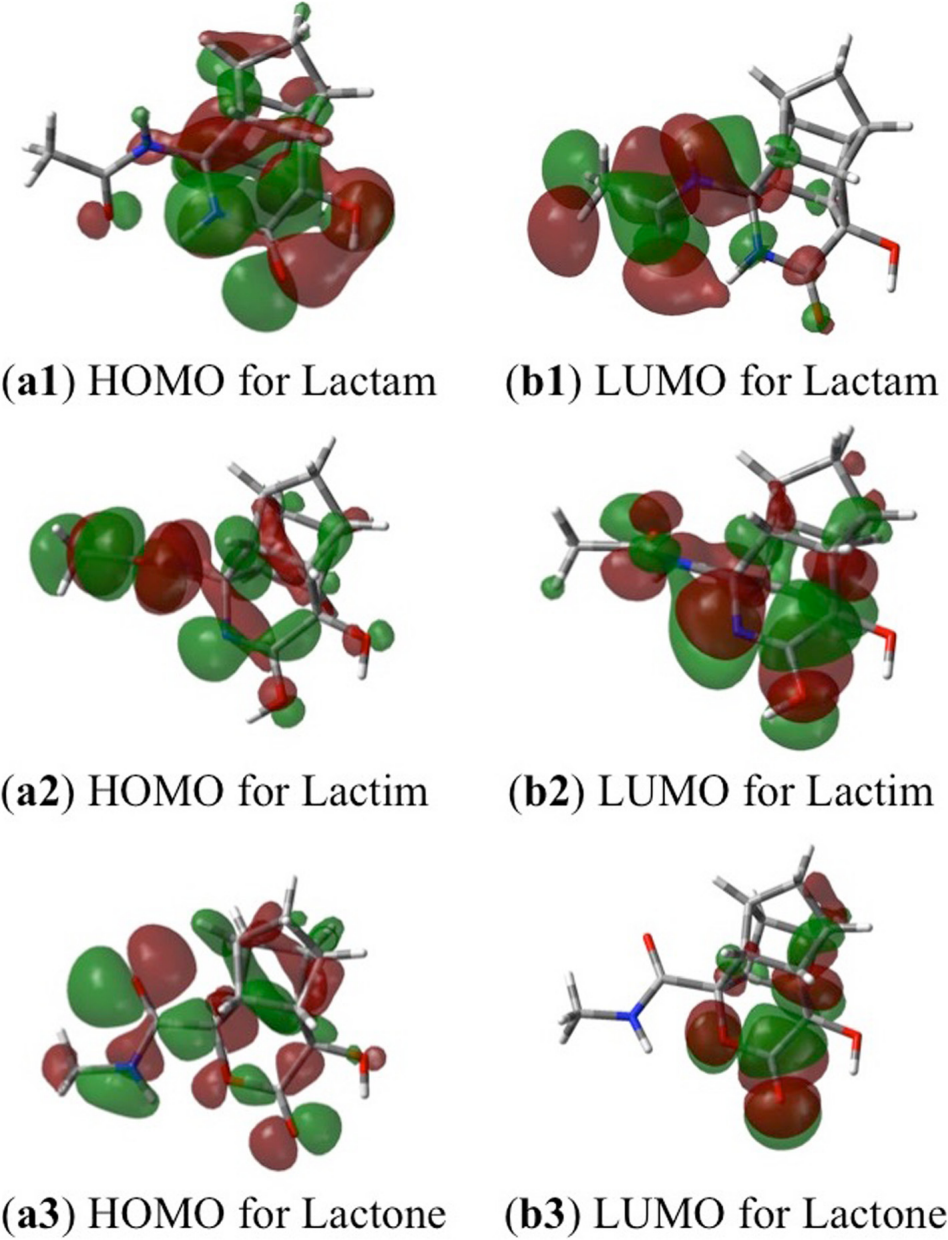
**The frontiers orbitals (HOMO and LUMO) of PCU-lactam (a1 and b1), PCU-lactim (a2 and b2) and PCU-lactone (a3 and b3) models [B3LYP/6-311G(d,p)].**

For more information about the electronic character of these compounds, the frontier orbitals (HOMO and LUMO), polarizability and dipole moments of PCU-lactam, lactim and lactone models were calculated next. The HOMO and LUMO frontier molecular orbitals are demonstrated in Figure 7.

It is evident that HOMO orbital of the lactam functional group is larger in comparison to corresponding orbitals located around the lactone and lactim cases. The size of the HOMO orbitals (which is an indication of the electron density) of the PCU-lactam and lactone models can be rationalized in terms of the corresponding experimental binding affinities. The lactam model exhibits larger electron density, which can potentially result into a better electrostatic (*i.e.* hydrogen bond) interaction between the substrate and the Asp25/Asp25′ groups. The LUMO around lactim functional group appeared to be most prominent of the three compounds.

Polarizability of a molecule is defined as the ease of distortion of the electron cloud of a molecular entity by an electric field (such as when in close proximity of a charged reagent). It is experimentally measured as the ratio of induced dipole moment (μ_ind_) to the field E which induces it. The dipole moment is the first derivative of the energy with respect to an applied electric field. It is used as a measure of the asymmetry in the molecular charge distribution. Much effort has been made to look for a correlation between the electric dipole moment of drug-like compounds and their biological activity [96-98] which requires the search for such a correlation between electron density distribution in a molecule of a given compound and its activity [44].

The polarizability and dipole moments for these compounds were calculated and is reported in Table 6.

**Table 6:** **Polarizability (Å** ^**3**^ **), dipole moment (Debye) and Gibbs free energy of solvation ΔG**_**solv**_ **(kcal/mol) values of the PCU-lactam and PCU-lactone models [B3LYP/6-311G(d,p)]**

**Compound**	**α (Å** ^**3**^ **)**	**μ (Debye)**	**ΔG**_**solv**_
			**(kcal/mol)**
**PCU-lactam**	81.218	6.4726	−17.925
**PCU-lactim**	81.663	3.5616	−17.085
**PCU-lactone**	79.324	1.2617	−8.893

∆G_solv =_ Gibbs free energy of solvation, α = Polarizability, μ = Dipole moment.

The higher values of both polarizability and dipole moment as well as the more negative Gibbs free energy of solvation, ∆G_solv_ for the PCU-lactam in comparison to the lactone model (Table 6), clearly confirms the more polar character of the PCU-lactam model. This characteristic appears to contribute towards a higher binding affinity of the peptide derivatives. The more negative MMPB(GB)SA binding free energy of solvation, ∆G_bind_ for the PCU-lactam in comparison to the lactone model Tables 1 and 2 is consistent with the more negative Gibbs free energy of solvation, ∆G_solv_ for the PCU-lactam in comparison to the lactone model reported in Table 6.

## Conclusion

A series of three novel PCU-lactone-CO-EAIS peptides were synthesized and tested for HIV-protease activity. Two of them exhibited significant activities (~1 μM). The most active inhibitor amongst these synthesized PCU-lactone peptides was the PCU-lactone peptide **9** (IC_50_ ~ 0.80 μM) with the AIS side chain*.* Comparison of the MM-PB (GB) SA binding free energy data of this compound in lactone series with the one of the most active PCU-lactam-NH-EAIS peptide (IC_50_ = 0.076 μM) that was synthesized before in our laboratory, reflect a higher binding affinity of the PCU-lactam peptide peptides against South African HIV-protease than the lactone series. This result supports the experimentally observed trend for HIV-PR IC_50_ values of the PCU-lactam-NH-EAIS inhibitor (IC_50_ = 0.076 μm) versus the lactone-peptides (IC_50_ = 0.850 μm).

From an electronic structure standpoint, a relatively more negative atomic charge was observed on the oxygen and nitrogen atoms of cage lactam model in comparison with cage lactone. The NQR results revealed higher delocalisation of charge distribution around oxygen atoms (O_2_ and O_3_) of the PCU-lactam, which can potentially react better with the active catalytic aspartic acid residues of HIV PR. It appears that the higher charge density, polarizability and the dipole moment due to the hetero-atoms of cage-lactam plays an essential role in their higher experimental activity and binding affinity than the corresponding cage-lactone peptides.

### Supplementary material

NMR spectra and HRMS data of all compounds are provided with the supplementary material. The 3D structures of all calculated compounds are also provided.

## Additional file

Additional file 1:
**Supporting Information.**

## Abbreviations

PIs: Protease inhibitors
HIV-PR: HIV protease
PCU: Pentacycloundecane
HCTU: 2-(6-Chloro-1H-benzotriazole-1-yl)-1,1,3,3-tetramethylaminium hexafluorophosphate
MM-PBSA: Molecular mechanics/poisson-boltzmann surface area
MM-PB(GB)SA methods: The molecular mechanics/generalized born surface area
