# Supplementary material for: Pentacycloundecane lactam vs lactone norstatine type protease HIV inhibitors: binding energy calculations and DFT study
Source: J Biomed Sci. 2015 Feb 18;22(1):15. doi: 10.1186/s12929-015-0115-5 (PMC4387594; doi:10.1186/s12929-015-0115-5)
Supplement: Additional file 1: — Supporting Information. [file 12929_2015_115_MOESM1_ESM.docx]

**Supporting Information**

Pentacycloundecane lactam vs lactone norstatine type protease HIV inhibitors: binding energy calculations and DFT study

Bahareh Honarparvar,^[a]^ Sachin A. Pawar,^[a]^ Cláudio Nahum Alves,^[b]^ Jerônimo Lameira,^[b]^ Glenn E. M. Maguire,^[a]^ José Rogério A. Silva,^[b]^ Thavendran Govender,^[a]^ and Hendrik G. Kruger,*^[a]^

^a^ Catalysis and Peptide Research Unit, School of Health Sciences, University of KwaZulu-Natal, South Africa

^b^ School of Molecular and Cell Biology, University of the Witwatersrand, Wits 2050, South Africa

^a^ Catalysis and Peptide Research Unit, School of Health Sciences, University of KwaZulu-Natal, South Africa

^b^ Laboratório de Planejamento e Desenvolvimento de Fármacos, Instituto de Ciências Exatas e Naturais, Universidade Federal do Pará, CP 11101, 66075-110, Belém, PA, Brazil

1. Material and Methods
2. ^1^H, and ^13^C Spectra’s of all compound
3. Table S1. The calculated ESP charges in vacuum and aqueous media.
4. **Material and Methods**

High resolution mass spectroscopic analysis was performed on a Bruker MicroTOF QII mass spectrometer in positive mode with an internal calibration. The peptides were purified on Shimadzu semi-preparative HPLC instrument with a flowrate of 17 mL/min on a Ace C18 (150 mm x 21.2 mm x 5 microns) with a UV/VIS detector (215 nm) and an automated fraction collector. A two-buffer system was employed, utilizing formic acid as the ion-pairing agent. Buffer A consisted of 0.1 % formic acid/H_2_O (v/v) and buffer B consisted of 0.1 % formic acid/acetonitrile (v/v).All ^1^H, and ^13^C NMR data were recorded on a Bruker AVANCE III 400 MHz spectrometer.

**General procedure for the synthesis of peptides using microwave power**

Stock solutions of all amino acids (0.2 mM), DIPEA (1 mM) and HBTU (2 mM) were prepared in DMF and peptides were synthesized on a 0.1 mmol scale on an automated CEM Liberty microwave peptide synthesizer. The resin bound peptide was removed from the peptide synthesizer and transferred to a manual peptide synthesis reaction vessel for the final cleavage. It was washed with DCM (3 x 10mL) and a cleavage mixture of 95:5% (v/v) TFA:DCM was added to the resin while nitrogen was bubbled through the solution for 1hr. The resin was washed three times with the cleavage mixture and the cleaved peptide was removed by filtration and collected in a flask containing water (100 mL). The filtrate was extracted several times with DCM so as to remove the peptide from the water layer. DCM was removed under reduced vacuum with at 40°C and the peptide remained as white powders were purified using semi-preparative HPLC.

**Figure 1** ^1^H NMR of **7**


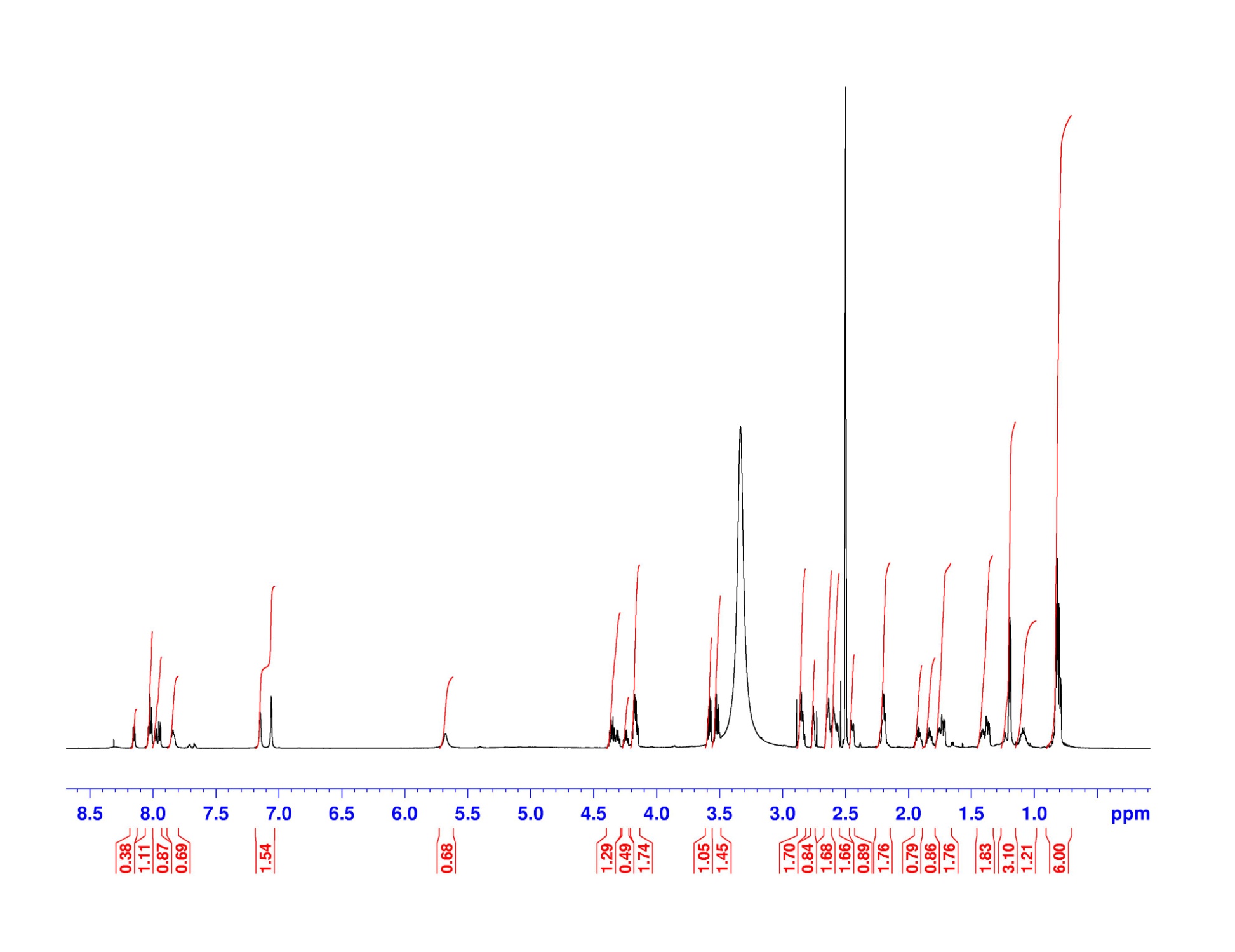

**Figure 2** ^13^C NMR of 7


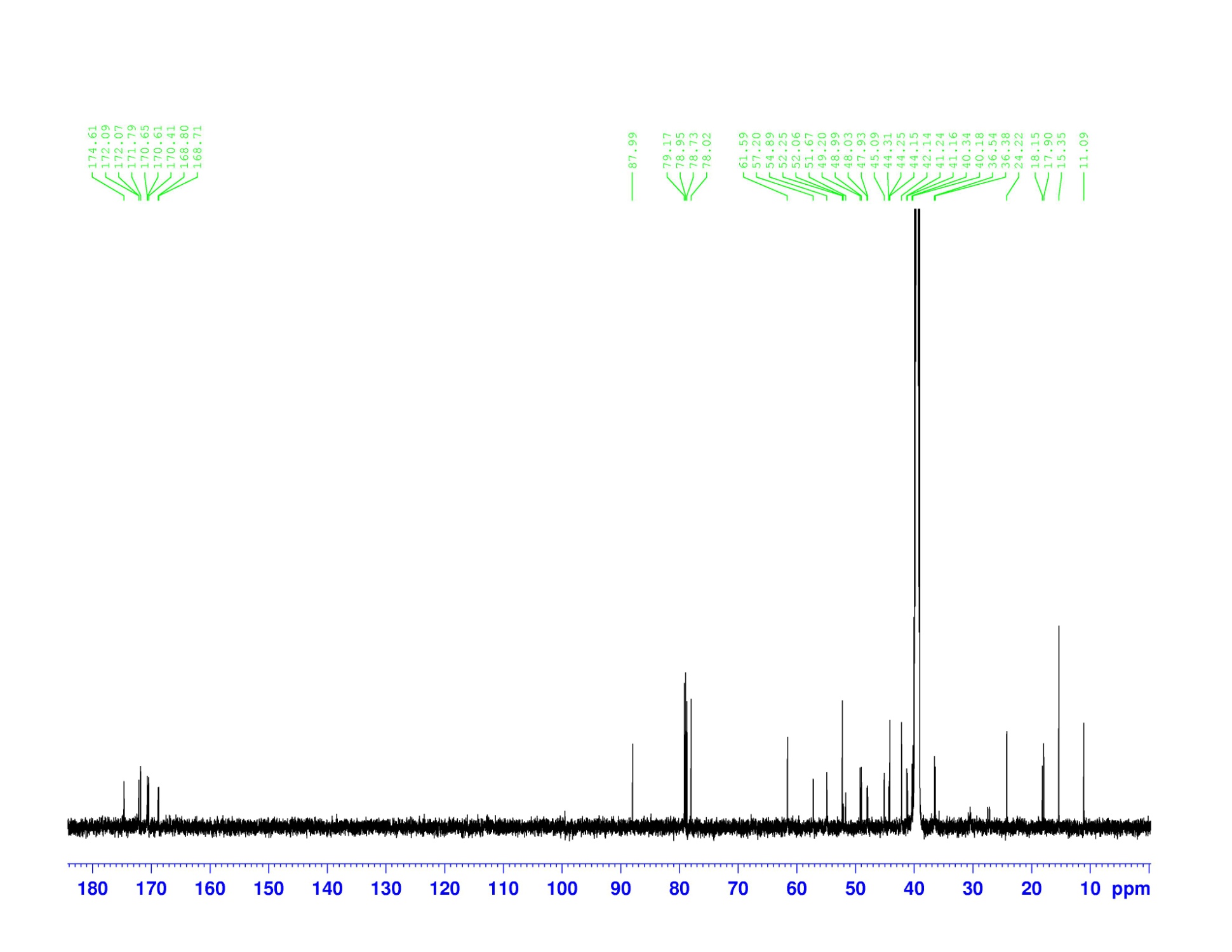

**Figure 3** HRMS of **7**

**Figure 4** ^1^H NMR of **8**


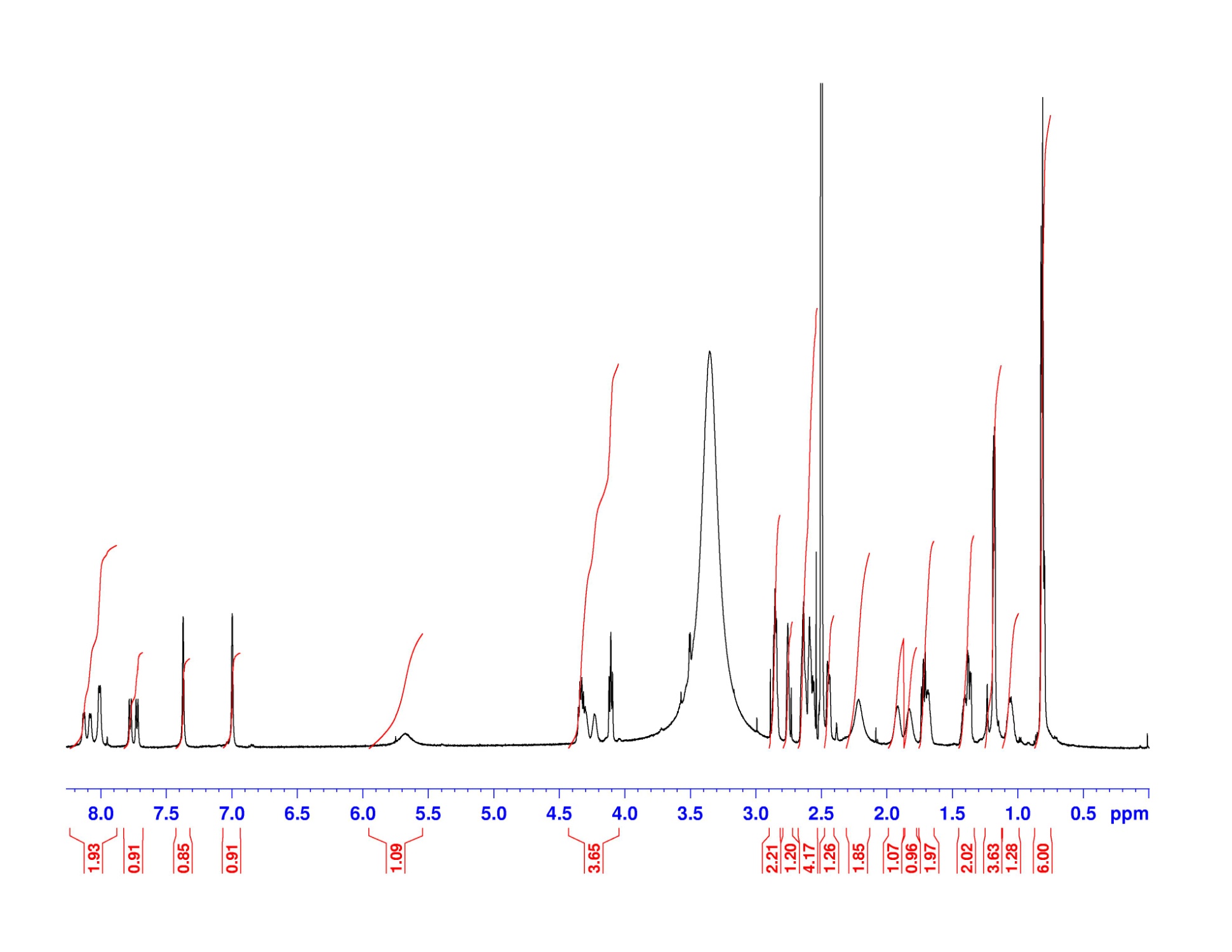

**Figure 5** ^13^C NMR of **8**


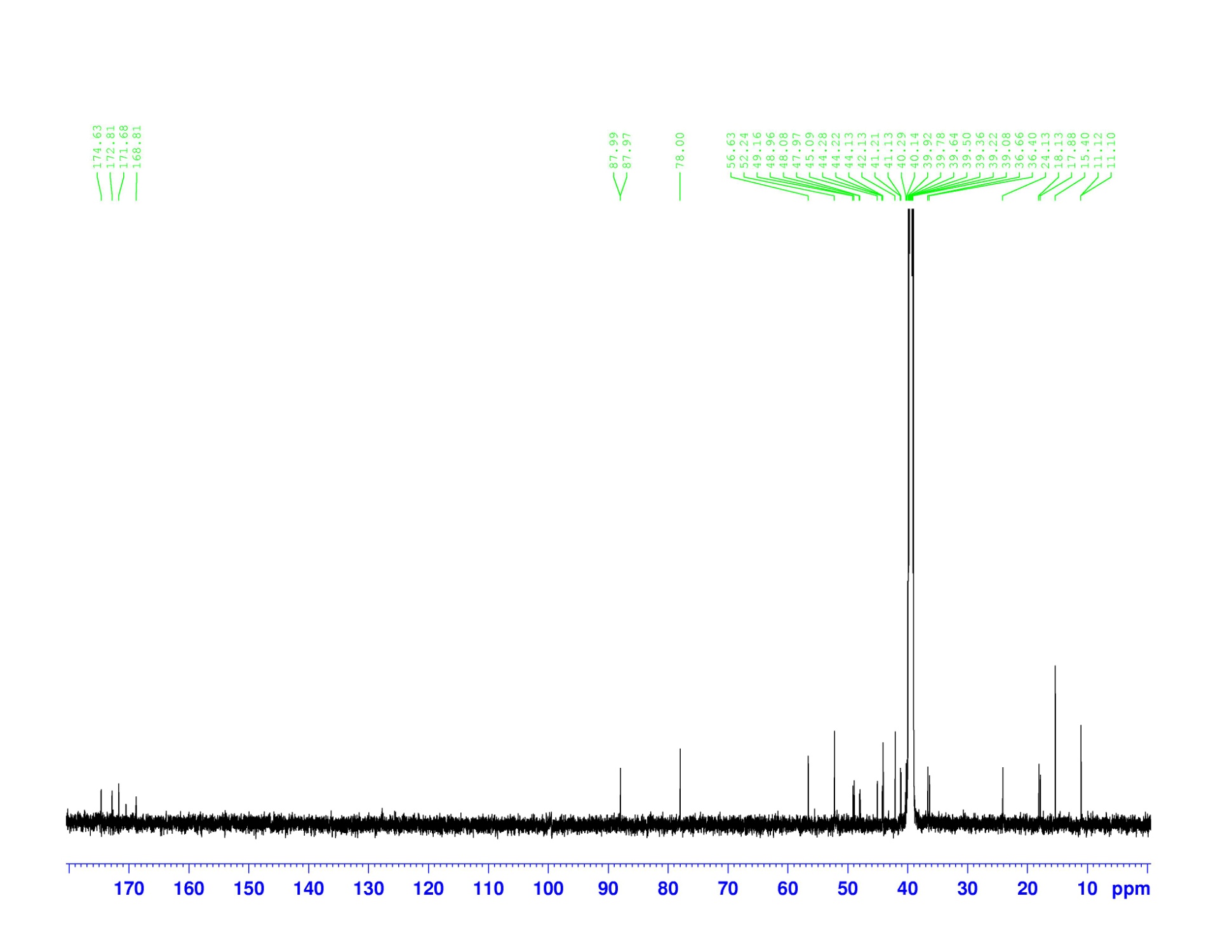

**Figure 6** HRMS of **8**

**Figure 7** ^1^ H NMR of **9**


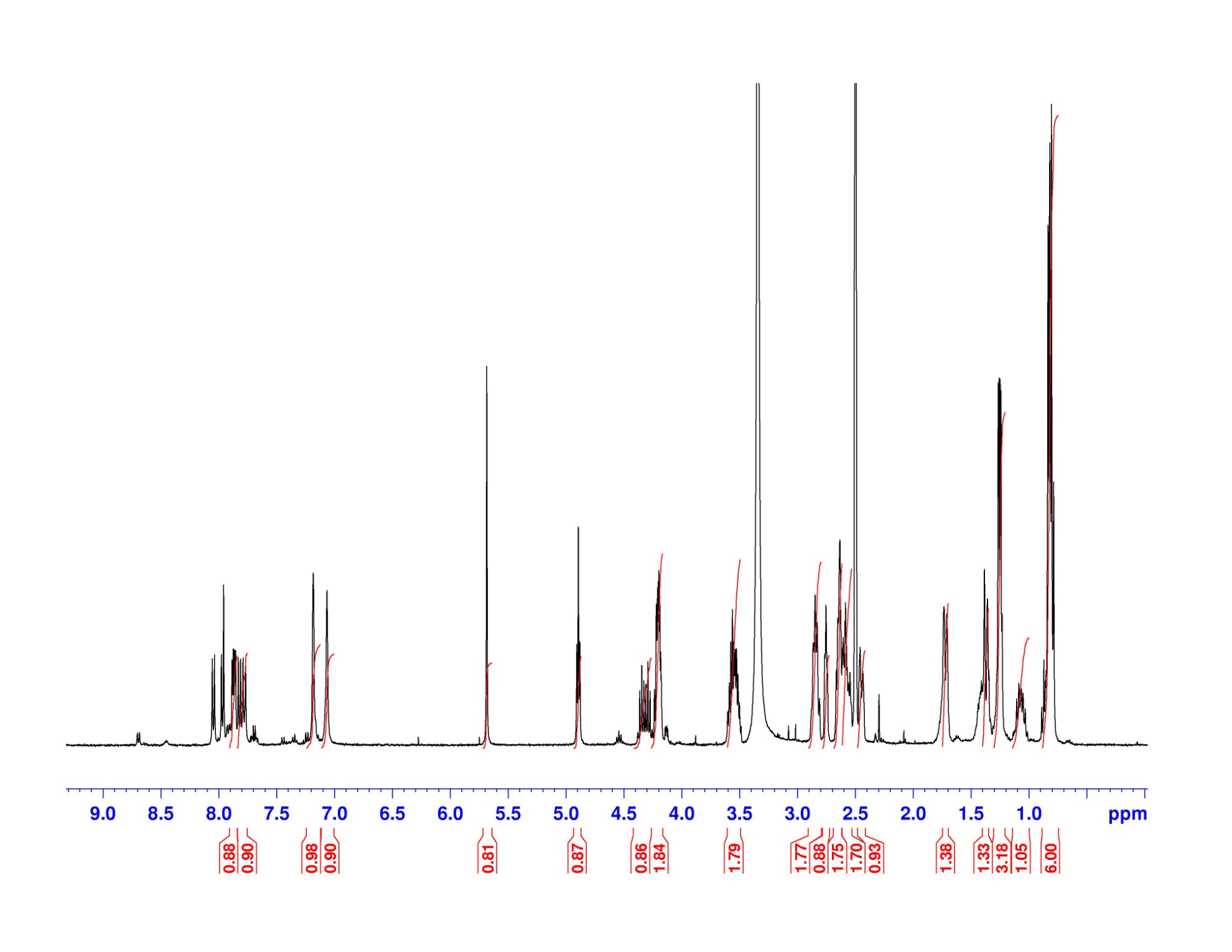

**Figure 8** ^13^C NMR of **9**


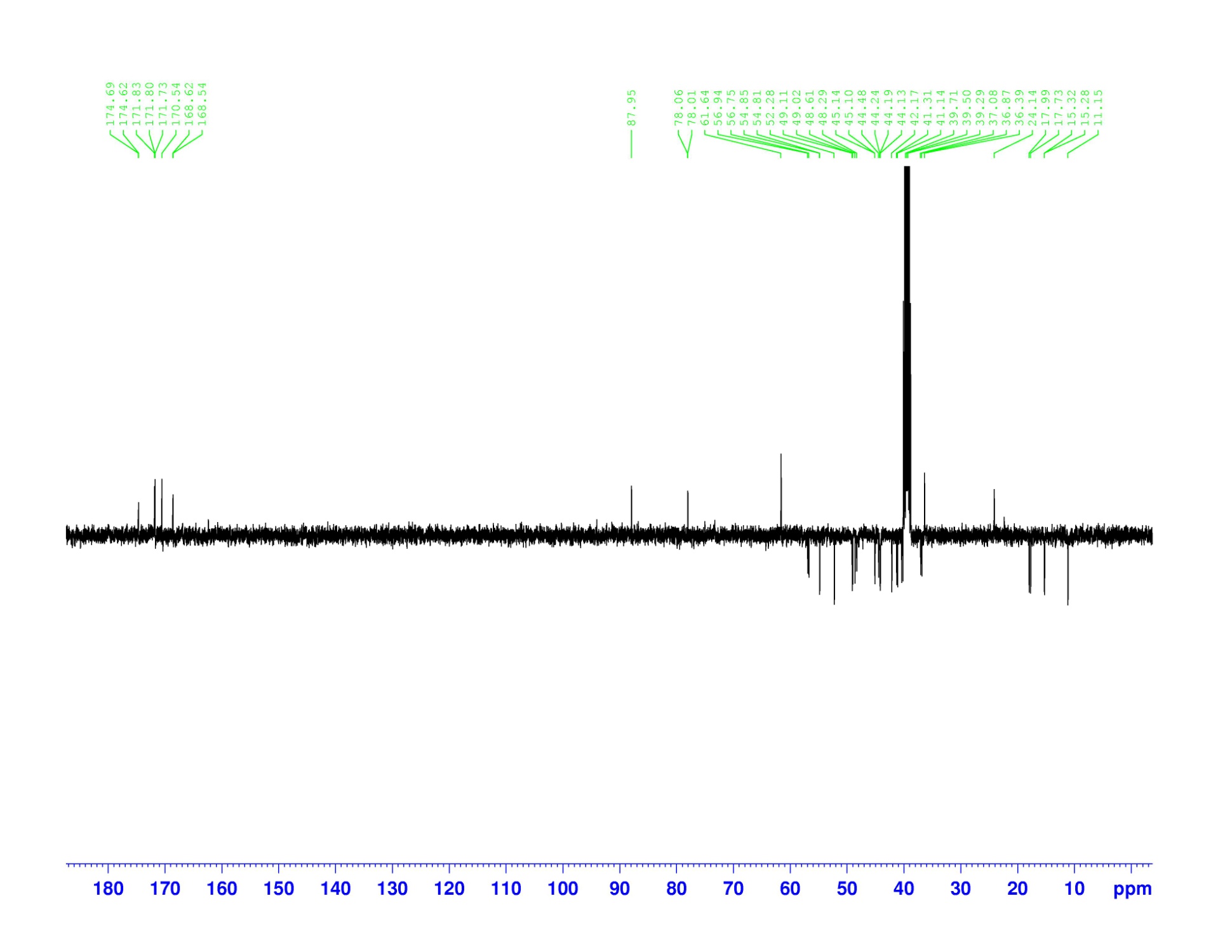

**Figure 9** HRMS of **9**

**Table S1.** The calculated ESP charges in vacuum and aqueous media [B3LYP/ 6-311G(d,p)].

| **Atom** | **Media** | **Lactam** | **Lactim** | **Lactone** |
| --- | --- | --- | --- | --- |
| **N_1_/O_1_** | **Vacuum**  **Water** | -0.743  -0.752 | -0.773  -0.849 | -0.457  -0.496 |
| **O_2_** | **Vacuum**  **Water** | -0.564  0.622 | -0.626  -0.637 | -0.528  -0.612 |
| **O_3_** | **Vacuum**  **Water** | -0.590  -0.653 | -0.614  -0.673 | -0.552  -0.616 |
| **N4** | **Vacuum**  **Water** | -0.692  -0.691 | -0.717  -0.736 | -0.659  -0.660 |
| **O_5_** | **Vacuum**  **Water** | -0.530  -0.629 | -0.729  -0.639 | -0.529  -0.610 |
